# Dynamic responses, GPS positions and environmental conditions of two light rail vehicles in Pittsburgh

**DOI:** 10.1038/s41597-019-0148-9

**Published:** 2019-08-12

**Authors:** Jingxiao Liu, Siheng Chen, George Lederman, David B. Kramer, Hae Young Noh, Jacobo Bielak, James H. Garrett, Jelena Kovačević, Mario Bergés

**Affiliations:** 1 Civil and Environmental Engineering, Carnegie Mellon University, 5000 Forbes Ave, Pittsburgh, PA 15213 USA; 2 Electrical and Computer Engineering, Carnegie Mellon University, 5000 Forbes Ave, Pittsburgh, PA 15213 USA; 3 Light Rail System, Port Authority of Allegheny County, 1000 Village Dr, Pittsburgh, PA 15241 USA; 4 Present Address: Mitsubishi Electric Research Laboratories (MERL), 201 Broadway, Cambridge, MA 02139 USA; 5 Present Address: McKinsey & Company Midwest, Blue Cross Blue Shield Tower, 300 E Randolph St #3100, Chicago, IL 60601 USA; 6 Present Address: Tandon School of Engineering, New York University, 6 MetroTech Center, Brooklyn, NY 11201 USA

**Keywords:** Civil engineering, Electrical and electronic engineering, Scientific data

## Abstract

We present DR-Train, the first long-term open-access dataset recording dynamic responses from in-service light rail vehicles. Specifically, the dataset contains measurements from multiple sensor channels mounted on two in-service light rail vehicles that run on a 42.2-km light rail network in the city of Pittsburgh, Pennsylvania. This dataset provides dynamic responses of in-service trains via vibration data collected by accelerometers, which enables a low-cost way of monitoring rail tracks more frequently. Such an approach will result in more reliable and economical ways to monitor rail infrastructure. The dataset also includes corresponding GPS positions of the trains, environmental conditions (including temperature, wind, weather, and precipitation), and track maintenance logs. The data, which is stored in a MAT-file format, can be conveniently loaded for various potential uses, such as validating anomaly detection and data fusion as well as investigating environmental influences on train responses.

## Abstract (summary)

**Table:** 

Design Type(s)	time series design • observation design • data collection and processing objective
Measurement Type(s)	public infrastructure • position
Technology Type(s)	Accelerometer • GPS navigation system
Factor Type(s)	temporal_interval • temperature • wind • weather • precipitation
Sample Characteristic(s)	Pittsburgh • anthropogenic habitat

Machine-accessible metadata file describing the reported data (ISA-Tab format)

## Background & Summary

The private freight rail industry in the U.S. makes $9.7 billion capital investment in maintaining the network, which is comprised of almost 140,000 miles of track and over 100,000 bridges in 2015^1^. However, in 2017, the Federal Railroad Administration still reported 11,699 train accidents/incidents including 1,223 derailments and 470 track-caused accidents/incidents in the nation^2^. To ensure safety and reduce maintenance cost, it is necessary to develop low-cost and reliable techniques to monitor the status of railroad networks continuously, especially track geometries. In practice, two traditional approaches are usually adopted to inspect track infrastructure: (1) visual inspection and (2) inspection using a dedicated track geometry car. Visual inspection is neither reliable nor convenient. While inspection using a dedicated track geometry car can provide accurate track geometry data, it requires interruptions of regular train operations, and each inspection session has a more expensive cost than visual inspection. Due to its high cost and interruptions, it is difficult to conduct frequent inspections using a track-geometry car. In recent years, researchers have proposed many indirect track inspection methods using sensors, such as accelerometers and GPS, installed on in-service trains for track geometry monitoring and change detection^3–11^ since it can be more reliable than visual inspection and costs less than inspection using a track-geometry car. Also, sensors installed on in-service trains can provide continuous monitoring of the track without affecting regular operations.

We monitored Pittsburgh’s light rail network from sensors placed on passenger trains, as a more economical monitoring approach than either visual inspection or inspection with dedicated track vehicles. Over time, we learned how the trains respond to each section of track and use a data-driven approach to detect changes to the track condition relative to its historical baseline. We instrumented one train in Fall 2013, and a second train in Summer 2015. We have been continuously collecting data on the trains’ position using GPS and their dynamic responses using accelerometers; in addition, our dataset includes environmental data as the trains were running on the track and the track maintenance logs from the light-rail operator.

Although there are some acceleration datasets for structure vibration testing^12^, human activity recognition^13^, senior fall detection^14^ and gait recognition^15^, at the time of writing, the DR-Train dataset is the only one to include multi-channel and high-frequency acceleration signals and GPS positions of light rail vehicles. The data were recorded from two light rail vehicles for four years with a variety of influential factors. This could be a benchmark dataset for comparing different vibration-based damage diagnosis algorithms. As a validation of our dataset, we have been able to detect changes in the tracks, which correspond to known maintenance activities. Besides detecting those changes, another usage of this dataset is for developing or validating data fusion methods. Data fusion methods integrate multiple data sources, such as multiple sensor channels on multiple light rail vehicles, to produce more consistent, accurate, and useful information than that provided by any individual data source. Our group has proposed a data fusion approach that integrates multiple accelerometers and GPS data sources from the same or different vehicles^11^. The DR-Train dataset has many other potential usages. For example, environmental factors of each service run are logged in the dataset; researchers can reuse the dataset to investigate influences of the weather and temperature on the dynamic response of the light rail vehicles.

In addition, there are many potential multi-disciplinary and interdisciplinary utilizations of the DR-train dataset. For instance, the vehicle is becoming a cyber-physical system that has more functions beyond a machine transporting people and goods. The light rail network can be considered as an urban sensing platform, and the DR-train provides a dataset that can be used for other applications, such as

- using mobile sensing to understand and monitor urban climate^16^;
- providing information of the light rail network for improving urban mobility^17^ and evaluating infrastructure resilience^18^;
- protecting and enhancing the infrastructure for ensuring railroad safety^9,19^;
- analyzing dynamic response of in-service LRVs for studying driver behavior characterization^19^.

## Methods

We use a data management system to collect and process dynamic responses and GPS positions of two passenger vehicles. We first introduce our monitoring target, the Pittsburgh Light Rail system, and our monitoring carrier, light rail vehicles in the following subsection.

### The Pittsburgh Light Rail and instrumented Light Rail Vehicles

The Pittsburgh Light Rail, called the ‘T’ lines, is a 42.2-km light rail network in Pittsburgh, Pennsylvania. This network is owned and operated by the Port Authority of Allegheny County (PAAC). It has 53 stations and around 28,000 daily ridership. The rail system, including imbedded street running track, direct fixation track and ballasted track, in this network uses the Pennsylvania Trolley gauge rail whose track gauge is 1,588 mm. Also, the network contains bridges, viaducts, and tunnels, and is exposed to variable environmental conditions. For example, the temperature we observed ranges from −20 °C to 35 °C. The variety of influential factors makes it a viable test-bed.

A light rail vehicle (LRV) is a standardized vehicle for U.S. cities. LRVs of the Pittsburgh’s light rail network have two models: Siemens SD-400 LRVs were built from 1985 to 1987 and assigned fleet numbers 4201 to 4255 after a mid-life overhaul by Construcciones y Auxiliar de Ferrocarriles S.A. (CAF) between 2004 and 2008. CAF also provided new LRVs which were built from 2004 to 2005 and assigned fleet numbers 4301 to 4328. Those LRVs are supplied by a 650 voltage direct current electrification system. We installed accelerometers and GPS antennas on LRVs 4306 and 4313. Each LRV has two motor trucks at opposite ends and a non-powered center truck below the articulation with a total length of 25.81 m, empty weight of 45 metric tons, total passenger capacity of 264 and a maximum speed of 80 km/h. The LRVs are typically run coupled together as two car trains during rush hour service.

### Data management system

**Fig. 1:**
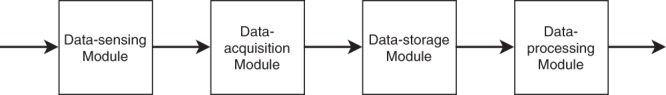
Proposed data-management system.

Our data management system consists of four key modules (as shown in Fig. 1): Sensing module, data-acquisition module, data-storage module, and data-processing module.

### Sensing module

**Fig. 2:**
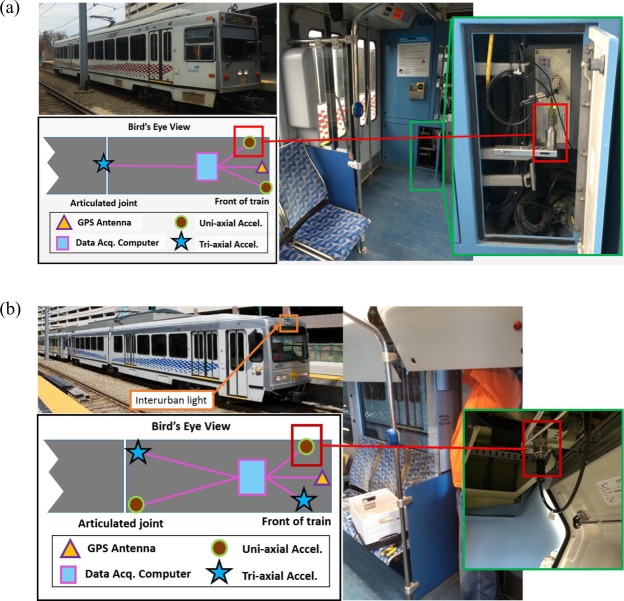
Data collection system of LRV 4306 and LRV 4313. For each subplot, top left picture shows the external view of the LRV. Bottom left figure shows a schematic of the sensor locations on the LRV, and the inside view of the train and one highlighted uni-axle accelerometer are shown in the right hand side pictures. (Figures from paper^11^ are reused here with license).

Figure 2 shows our instrumentations of two LRVs (fleet number 4306 and 4313). In 2013, we instrumented the LRV 4306 by placing two uni-axial accelerometers inside the cabin of the train (VibraMetrics 5102^20^) and a tri-axial accelerometer (PCB 354C03^21^) on the central wheel truck. The central wheel truck, or the central bogie, is a chassis attached to a vehicle and carrying wheelsets with a suspension system. Since it is not powered, the electrical noise is minimized. However, sensing system on the central wheel truck has higher installation and maintenance costs than the system inside the cabin. For the second LRV instrumented (fleet number 4313) in 2015, we placed more sensors, including two uni-axial accelerometers (VibraMetrics 5102) and two tri-axial accelerometers (PCB 354C03) inside the train, for improving the system. To collect the position data, we placed a low-cost BU-353 GPS^22^ antenna on the LRV 4306 and 4313. The GPS antenna in the LRV 4313 was installed within the interurban light enclosure for having a view of the sky. Tables 1 and 2 show important specifications of those sensors.

**Table 1:** Operating specifications of accelerometers.

Type	Model	No. of axles	Sensitivity	Amplitude range	Resonant Frequency
Piezoelectric Accelerometers	Vibra Metrics 5102	1	500 mV/g (±5%)	±10 g	2.5 kHz
	PCB 354C03	3	500 mV/g (±10%)	±50 g	≥12 kHz

**Table 2:** Operating specifications of GPS receivers.

Model	Number of channels	Sensitivity	Update rate	Accuracy
BU-353	48	−163 dBm	1 Hz	<2.5 m

### Data-acquisition and data-storage module

For data acquisition, we connected National Instruments USB-powered data acquisition hardware to a computer. Our data acquisition system samples acceleration signals at 1.6KHz and GPS position at 1 Hz and logs the data to an external hard drive. We download the data manually from the onboard computers to our local computer every two weeks, because we are not allowed to install wireless devices on LRVs. We use National Instruments LabVIEW^23^ to control the hardware and acquire acceleration data.

### Data-processing module

**Fig. 3:**
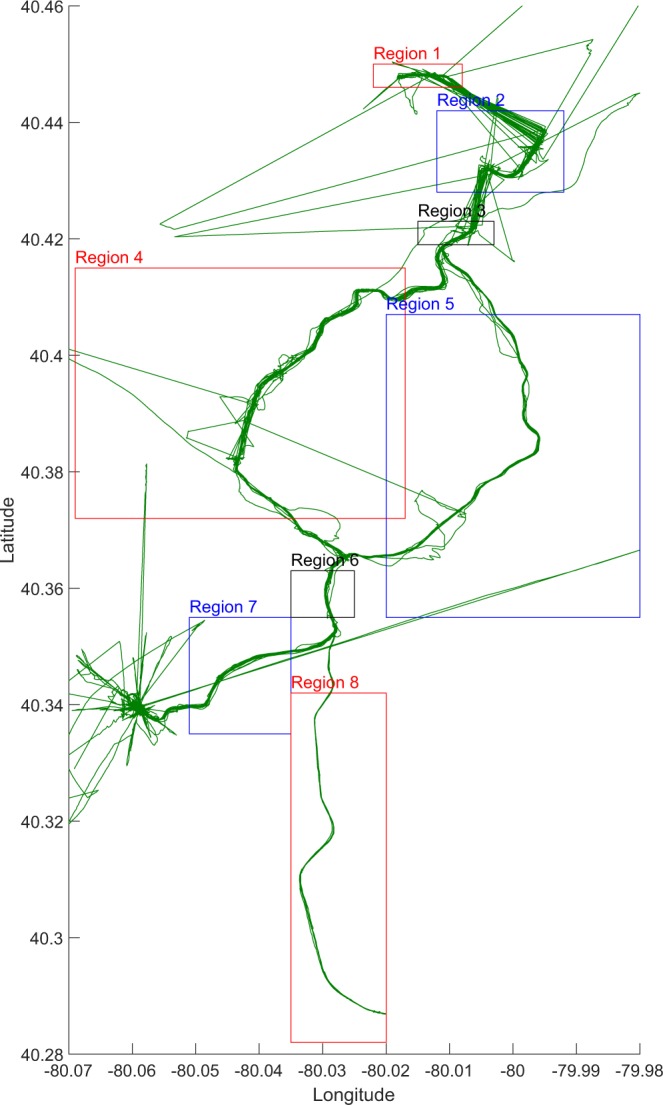
An example of the GPS trace of several passes through the ‘T’ lines and the associated track regions used for analysis.

Before analyzing the collected data, it is necessary to segment it by geographical region. There are two reasons: first, we want to have the LRVs travel along the same path in each particular region; second, the GPS signal will be lost in tunnels, and it is difficult to determine the location of excitations. The rail network is divided into eight regions to ensure continuous GPS trace in each region. Figure 3 shows the GPS trace of several passes and the associated track regions.

**Fig. 4:**
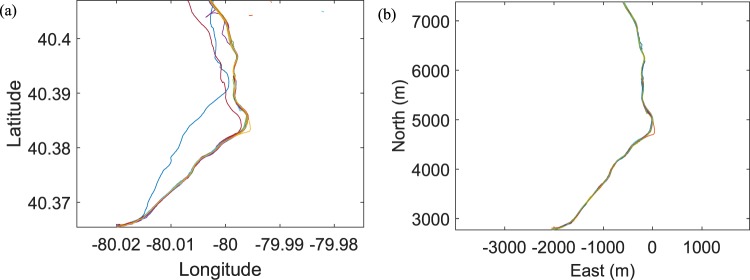
The lefthand and righthand figures show GPS traces of 40 different outbound passes in the 5th track region before and after registration using ICP, respectively.

The next step in processing the GPS data is to register it to a ground truth of the track position because the measurement error from collected GPS data could cause misalignment among different passes. PAAC provided us the foot by foot GPS position data, which allowed us to achieve this registration. We first utilized the iterative closest point (ICP)^24^ algorithm to eliminate the global mismatch of the GPS data by minimizing the difference between every two GPS point clouds. Figure 4 show the GPS traces of 40 different outbound runs in the 5th track region before and after registration using ICP, respectively. For the local mismatch, the one-nearest neighbor algorithm is applied to register GPS position data of different runs to the nearest point of the ground truth GPS.

We also present environmental conditions and maintenance schedules during this monitoring period in the following sections.

### Environmental conditions

Environmental conditions, including temperature, wind, weather, and precipitation, vary significantly when we collect the data. It is not only because all the sensors are sensitive to the operating temperature, but also steel rail expands as it heats up. To record the environmental conditions, we used the time stamp and the trains’ GPS position to query environmental conditions from a weather data provider called Forecast.io when we were processing the collected data. This weather data provider gathered hour-by-hour environmental observation from tens of thousands of stations worldwide including the PAAC rail service system territory.

### Maintenance schedules

Because we were not allowed to conduct an experiment on the track, we had to wait for changes made by PAAC’s track maintenance. PAAC provides us with track allocation reports, which are stored in the DR-Train dataset as well. Those reports allow us to calibrate our data collection system and validate our track monitoring approaches.

### Known issues

- Even after GPS registration using ICP and one-nearest neighbor algorithms, GPS position uncertainties, which cause misalignments of accelerations in the spatial domain, still exist. This is because the GPS receiver has a 2.5-meter accuracy on average and has variable accuracy levels at different locations. The misalignments can be illustrated by a 20% classification error reduction for track change detection before and after alignment and post-processing as presented in the work of Lederman *et al*.^10^. In the paper, Lederman *et al*. enforced sparsity of track profile bumps to overcome the position uncertainty from GPS error. The location of sparse bumps is used to align accelerations, and the height of sparse bumps is used as a measure of the track height and indicates changes in the rail track.
- We found that the train’s ventilation system is a source of noise. On a warm day, the air conditioner turns on, and there is higher energy at 30 Hz. Whereas on a cold day, the air conditioner turns off, so we observed less energy at 30 Hz. The signal energy at 30 Hz does not depend on the train speed or the track roughness.
- Individual sensors or sensor channels can be malfunctioning. Also, the likelihood of any sensor malfunctioning increases with increasing number of sensors. To address this problem, our group has developed a data fusion approach^11^. The approach helps to combine raw data (or features extracted from raw data) that is collected from different sensors and from multiple trains for producing more reliable and accurate information than that provided by any individual sensor channel. Specifically, it first approximately aligns acceleration signals of different sensor channels by calculating cross correlations and then applies an adaptive Kalman filter that weighs acceleration data according to its estimated reliability.

## Data Records

From 2013 to 2016, we collected acceleration and GPS position data with corresponding environmental conditions and maintenance logs. The DR-Train dataset includes 31 months of data from LRV 4306 and 11 months of data from LRV 4313. Table 3 shows the number of passes collected from the eight geographical regions mentioned in the Method section. ‘Inbound’ means that the LRV goes from South to North, and ‘outbound’ means the opposite direction. In some regions, the number of outbound passes is larger than that of inbound passes because, as the train emerges from the tunnel, there is a delay before the GPS can get a position lock.

**Table 3:** Number of passes collected from LRVs 4306 and 4313 through the eight geographical regions.

Region	LRV 4306		LRV 4313		
	Inbound	Outbound	Inbound	Outbound	Total
1	226	363	51	82	722
2	569	577	138	136	1420
3	579	567	135	131	1412
4	288	292	33	34	647
5	317	317	102	96	832
6	440	425	116	110	1091
7	356	342	85	80	863
8	180	182	65	63	490
Total	2955	3065	725	732	7477

All the data details and directories are stored in ‘pass’ MATLAB objects in ‘\data_files\LRV4306’ and ‘\data_files\LRV4313’ folders. The ‘pass’ object is defined in the MATLAB class function ‘pass.m’. Properties and Methods of the ‘pass’ class are described in Table 4. For LRV 4306, there are 30,096 ‘pass’ objects stored in the file ‘\data_files\LRV4306\obj_dict.mat’. LRV 4306 has five acceleration channels (property ‘sensor’), corresponding to the two uni-axial accelerometers inside the train cabin and the three channels of the tri-axial accelerometer on the wheel truck. For LRV 4313, there are 18,929 ‘pass’ objects stored in the file ‘\data_files\LRV4313\obj_dict.mat’. LRV 4313 has 8 acceleration channels (property ‘sensor’), corresponding to the two uni-axial accelerometers and two tri-axial accelerometers inside the train cabin. The raw acceleration and GPS position data are stored in ‘acceleration_data’ and ‘gps_data’ folders. These data can be retrieved from files into the MATLAB/Octave workspace by loading acceleration filenames (property ‘acc_raw’) and GPS filenames (property ‘gps_raw’). Each raw acceleration file only has a one-column MATLAB matrix in double-precision type, which is the logged temporal acceleration data of one single channel during one service run. Each raw GPS file has a five-column MATLAB matrix in double-precision type, corresponding to the longitude, latitude, altitude, velocity and time stamp during one service run. The time stamp is a serial date number that represents the whole and fractional number of days from a fixed, preset date, January 0, 0000, in the proleptic ISO calendar.

**Table 4:** Properties and methods of the ‘pass’ class.

	Name	Description
Properties	acc_al	address where aligned accelerometer data is stored
	acc_raw	address where accelerometer data is stored
	acc_samp	accelerometer sampling rate (Hz)
	count	this is the indice of the pass in terms of all passes
	date	date in UTC time
	date	date string in local time
	daten	property date in ‘datenum’ format
	datenl	property datal in ‘datenum’ format
	direction	‘inbound’ our ‘outbound’
	gps_al	address where aligned GPS is stored
	gps_raw	address where GPS is stored
	gps_samp	gps sampling rate (Hz)
	region	region on the map where the signal comes from
	sensor	number of sensor channel
	summary	weather summary text at time of pass
	summary8	weather summary text 8 hours prior to pass
	temp	temperature at the time of the pass
	temp8	temperature 8 hours prior to pass
	train	number of the train
Methods	addlistener	Add listener for event.
	addprop	Add dynamic property to MATLAB object.
	coor	show the coordinate system for the selected data
	date_bounds	This function takes an array of passes, as well as lower and upper bounds for the dates (as strings), then plots the data that falls between the two speci_ed date strings. Note this outputs the dates selected.
	delete	Delete a handle object.
	eq	== (EQ) Test handle equality.
	findobj	Find objects matching speci_ed conditions.
	findprop	Find property of MATLAB handle object.
	ge	>= (GE) Greater than or equal relation for handles.
	gt	> (GT) Greater than relation for handles.
	isvalid	Test handle validity.
	le	<= (LE) Less than or equal relation for handles.
	listener	Add listener for event without binding the listener to the source object.
	lt	<(LT) Less than relation for handles.
	ne	=(NE) Not equal relation for handles.
	notify	Notify listeners of event.
	plot_both	Plot the data in time and frequency domain
	plot_freq	Plot the data in the frequency domain
	plot_gps	Plot the GPS trace on a map
	plot_time	Plot the data in the time domain
	scatter	Plot energy of the signal at the GPS points

Weekly maintenance schedule sheets from Light Rail System, PAAC are stored in ‘\data_files\track_maintenance_logs’ folder. Those files provide information on what was happening on the rail network. Typically, the only work, which matters for this research, is done by the ‘Way Department’ that maintains the tracks.

This dataset is available from the Zenodo repository (10.5281/zenodo.1432702)^26^.

## Technical Validation

We validated the technical quality of the presented dataset from three perspectives. First, we consider a series of possible failure situations for the process of data collection and justify why we can rule them out; second, we consider a series of basic requirements that a high-quality dataset should satisfy and validate that the presented dataset satisfies all the requirements; third, a series of works have shown that changes to the tracks can be successfully detected based on the presented dataset.

### Data collection failures

We consider the possible failure situations (missing data or corrupted data) as follows.

- The installed accelerometers fail to sense acceleration signals;
- The data-acquisition system fails to transfer acceleration signals to the installed computer;
- The installed computer fails to store the acceleration signals on the local disk;
- The data-processing system fails to organize acceleration signals correspondingly in the database.

**Fig. 5:**
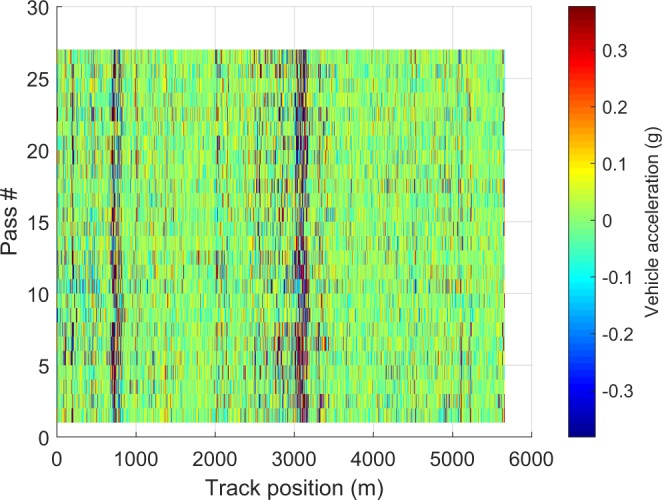
Visualizing acceleration samples in the spatial domain. We visualize the 27 acceleration passes of accelerometer channel five in region five during May 2014. Each horizontal line shows one vehicle acceleration record at different track positions in the selected region.

Data visualization is an efficient way to rule out the first and the second failure situations. We added chart blocks in our LabVIEW implementation to visualize the collected acceleration signals instantaneously. We also plotted the collected data on our local computer. Figure 5 shows 27 properly collected spatial-domain acceleration samples of accelerometer channel 5 in geographical region 5 (outbound direction) during May 2014. In the supplementary documentation, we also present spatial-domain acceleration signals of accelerometer channel 5 of LRV 4306 in other geographical regions (outbound direction) during the year of 2015. By checking the size of logged data directly, we can rule out the third failure situation. Also, for this dataset, we rule out the top three failures by checking whether signals are sampled continuously with a constant sampling rate in the time domain. Specifically, we first ensure that there are no Null values in the collected signals and check whether the calculated duration (divide the total number of samples by the sampling rate) is equal to the measured duration (last timestamp minus the first timestamp). To rule out the fourth failure situation, we have to download the data back to our local computer and validate the technical quality of them after data processing. The following section introduces two basic requirements of a high-quality dataset and their validations.

### Basic requirements

We consider the basic requirements that a high-quality dataset should satisfy.

**Fig. 6:**
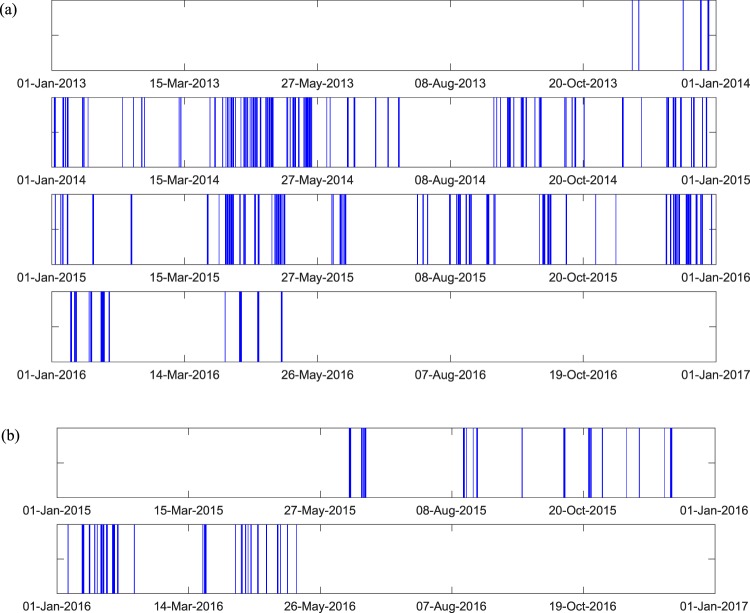
The top and bottom figures show time periods when sensors on LRV 4306 and LRV 4313 were recording, respectively. Blue lines indicate days when accelerometers and GPS were recording. The causes of recording gaps are explained in section Basic requirements.

- R1: The acceleration signals collected from the same accelerometer channel should be consistent across trials during a period, such as one day, one week or one month. Since the acceleration signal of each trial reflects the roughness of the same track, the overall profiles of acceleration signals should be similar across trials. Otherwise, this dataset is problematic;
- R2: The acceleration signals should be correlated with the GPS positions. As a discrete-space signal, the acceleration signal of each trial is associated with the position of the track. For example, amplitude of the acceleration signal around the train station should be low. Otherwise, this dataset is problematic;

Figure 6 shows time periods (days) when sensors on LRVs 4306 and 4313 were recording. Blue lines indicate recording days, and white gaps indicate recording gaps in the dataset. The recording gaps are caused by one of three reasons: (1) Sometimes, the data-storage module runs out of space before downloading the data, and we are not allowed to install wireless sensors on the LRV in order to avoid interference between signals from our system and those of train control & communication systems; (2) The data management system may not restart automatically after restarting the light rail vehicles, although we have programmed it to do that; (3) The light rail vehicles were not in service because of maintenance, inspection and repair.

#### Validation of R1

To prove the consistency of acceleration signals from the same accelerometer channel, we use the two-sample Kolmogorov-Smirnov test (K-S test)^27^. The two-sample K-S test is a general nonparametric method for comparing two samples. It quantifies a distance between the empirical distribution functions of two samples. The Kolmogorov-Smirnov statistic is

$${D}_{n,m}=\mathop{{\rm{\sup }}}\limits_{x}| {F}_{1,n}(x)-{F}_{2,m}(x)| ,$$

where *F*_1,*n*_ and *F*_2,*m*_ are the empirical distribution functions of the two samples, respectively, and sup is the supremum function. The null hypothesis that the samples are drawn from the same distribution is rejected at level *α* if

$${D}_{n,m} > c(\alpha )\sqrt{\frac{n+m}{nm}},$$

where *n* and *m* are the sizes of the two samples respectively, and for 5% and 1% rejection levels, *c*(*α*) is equal to 1.36 and 1.63, respectively.

**Fig. 7:**
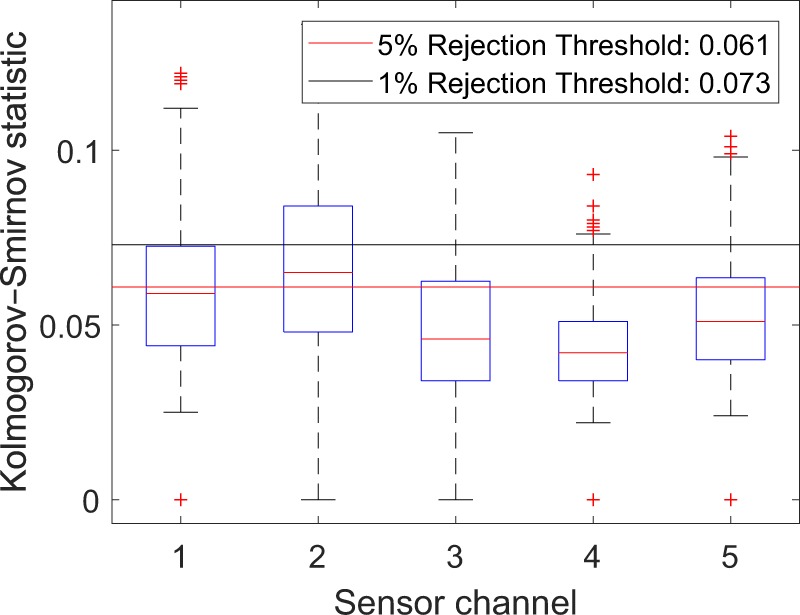
A result of the two-sample K-S test. For LRV 4306, there are 27 outbound trails during May 2014 in region five. We randomly sample 1,000 acceleration amplitudes from each trail and calculate the K-S statistics of each pair of samples. If the statistic is above the red line, the null hypothesis that two samples are drawn from the same distribution is rejected at level 5%. If the statistic is above the black line, the null hypothesis is rejected at level 1%.

The null hypothesis of our test is that the acceleration signals from the same sensor channel in the same time period and the same geographical region are drawn from the same distribution. The boxplot (Fig. 7) shows a result of this test for LRV 4306. We first resample the time-domain signal in the spatial domain and randomly sample 1,000 acceleration amplitudes from each trail of the same sensor channel of LRV 4306 during May 2014 in region five. LRV 4306 ran 27 outbound trails during May 2014 in region five. We then calculate the K-S statistics of each pair of acceleration samples. For the tri-axle accelerometer installed on the central wheel truck (less electrical noise), the tests of the three channels are not rejected, both at 5% significance level and at 1% significance level. However, the two uni-axle accelerometers installed in the cabin are rejected at 5% significance level, but not rejected at 1% significance level.

#### Validation of R2

**Fig. 8:**
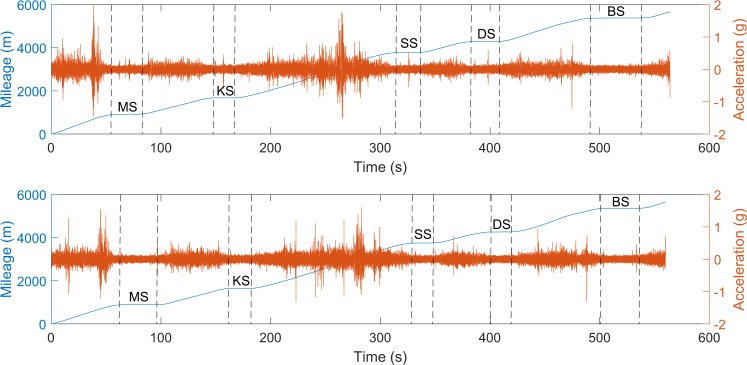
Two examples of acceleration and position data in region five. When the LRV was at those five stations: Memorial Station (MS), Killarney Station (KS), South Bank Station (SS), Denise Station (DS), and Bon Air Station (BS), the amplitude of the acceleration signal is small, and mileage stays the same.

Because the trains were moving with different speeds, it is difficult to prove that every single acceleration and GPS position are correlated in the time domain. However, we can prove the geographical alignment of acceleration signals and GPS positions with the information of train stations. When arriving at a train station, the LRV stays stationary and idles. We assume that amplitude of the idling accelerations would be lower than the traveling accelerations due to the excitation of the tracks. Figure 8 shows two examples of acceleration and position data in Region 5. There are five stations in Region 5: Memorial Station (MS), Killarney Station (KS), South Bank Station (SS), Denise Station (DS), and Bon Air Station (BS). We can observe that the amplitude of the acceleration signal is small when the LRV was at stations, and the mileage stays the same at stations.

### Publications based on the presented dataset

Papers^9–11,28^ using the data described here have been published. Lederman *et al*. used the collected dataset to detect changes in the tracks, including changes in the tracks due to repair and changes in track geometry due to tamping, by applying implicit and explicit models. The implicit model first extracts different features from the raw acceleration signals and then performs change detection with some common methods, including cumulative sum chart control (CUSUM)^29^, generalized likelihood ratio (GLR)^29^ and Haar filter^30^. The explicit model solves for the parameters of the train’s main suspension by enforcing sparsity in modeling the train system and learns where in the tracks the train is most excited by enforcing sparsity in the track profile. Lederman *et al*.^11^ also proposed a data fusion approach for enabling data-driven rail-infrastructure monitoring from multiple in-service trains using the implicit model of the tracks.

## Usage Notes

The data is provided in a MAT-file format with query files, and therefore it is convenient to load it in MATLAB. The script file (\code\main_script.m) calls the function (\code\load_processing.m) for loading and processing data and returns ‘pass’ objects, acceleration signals, and GPS positions. The ReadMe file provides more information about the usage of the DR-Train dataset.

## Supplementary Information

### ISA-Tab metadata file

Download metadata file

### Supplementary information

Supplementary document

## Data Availability

The code used to register GPS positions via ICP can be downloaded from MathWorks’ file exchange^25^. The script and function used to load and process the data files can be downloaded from the Zenodo repository (10.5281/zenodo.1432702)^26^. The codes have been tested using MATLAB 2017 on a typical personal computer and can run using different MATLAB versions and computers.
