# Supplementary material for: Dynamic responses, GPS positions and environmental conditions of two light rail vehicles in Pittsburgh
Source: Sci Data. 2019 Aug 12;6:146. doi: 10.1038/s41597-019-0148-9 (PMC6690915; doi:10.1038/s41597-019-0148-9)
Supplement: Supplementary file 2 — Supplementary document [file 41597_2019_148_MOESM2_ESM.pdf]

Supplementary material:

This document presents uni-axial acceleration signals of LRV 4306 in different regions, 2015. It supports our response to the third comments of the first reviewer.

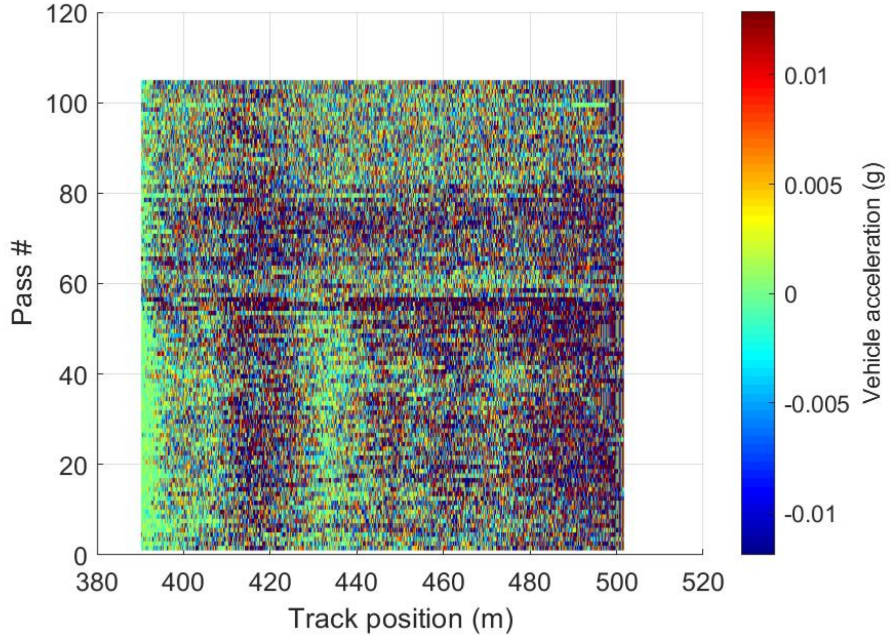

Figure A1: Uni-axle acceleration signals of LRV 4306 in region 1, 2015

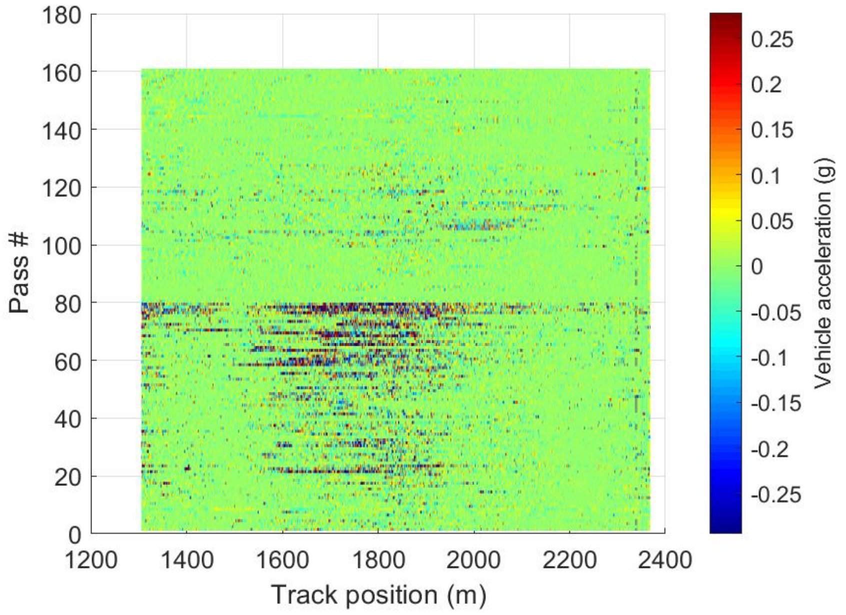

Figure A2: Uni-axle acceleration signals of LRV 4306 in region 2, 2015

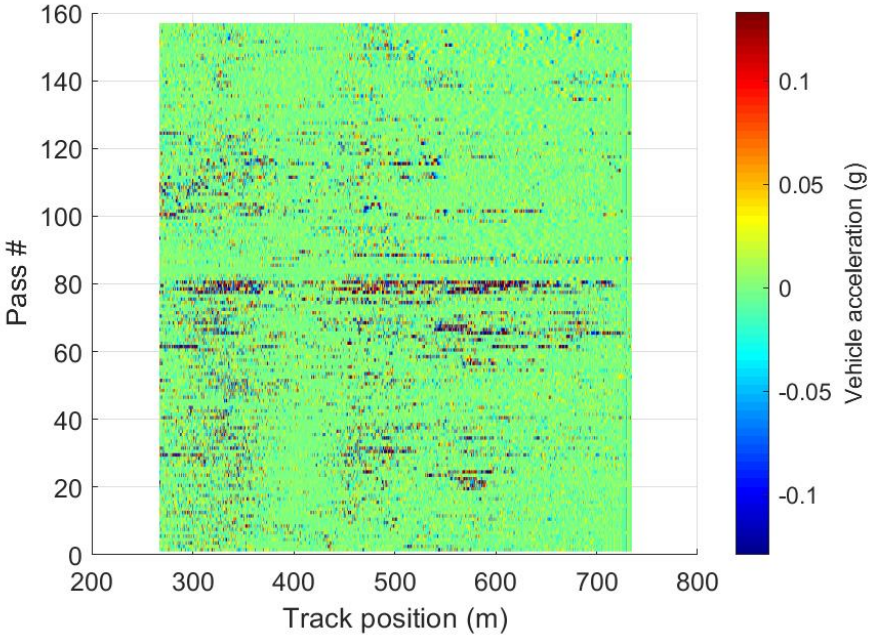

Figure A3: Uni-axle acceleration signals of LRV 4306 in region 3, 2015

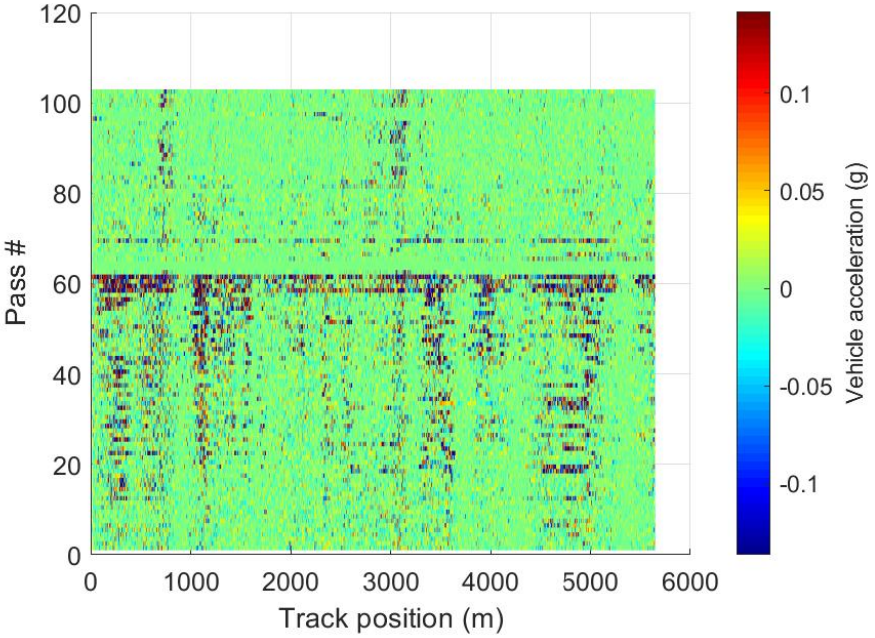

Figure A4: Uni-axle acceleration signals of LRV 4306 in region 5, 2015

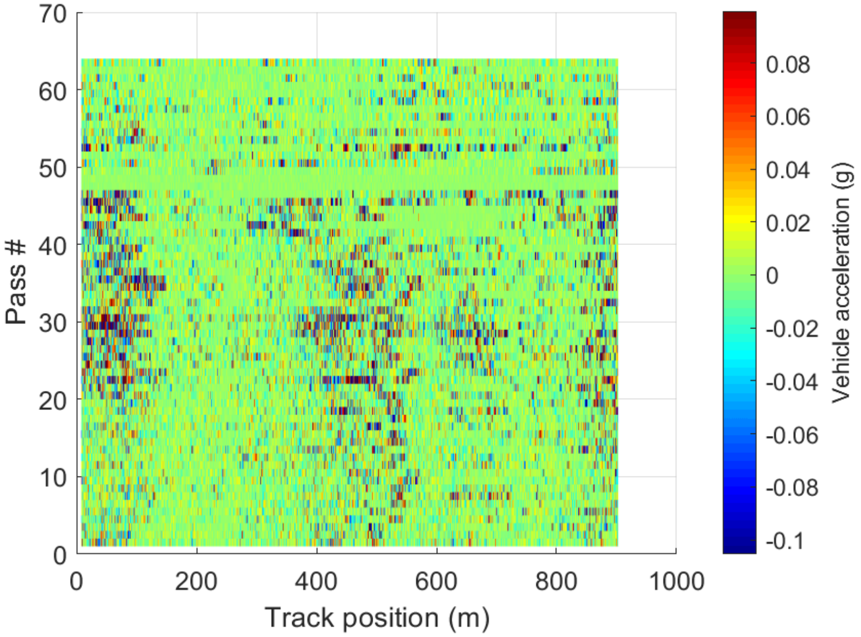

Figure A5: Uni-axle acceleration signals of LRV 4306 in region 6, 2015

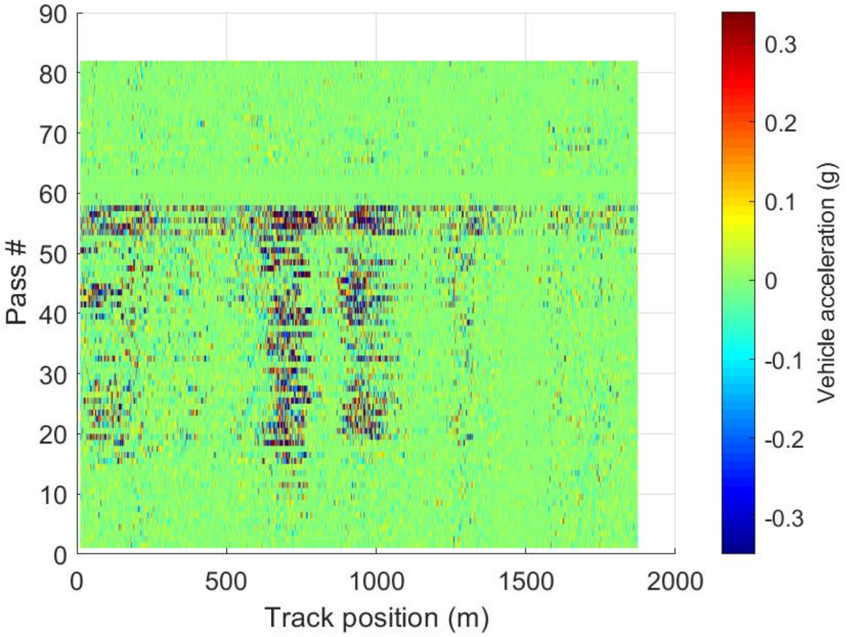

Figure A6: Uni-axle acceleration signals of LRV 4306 in region 7, 2015

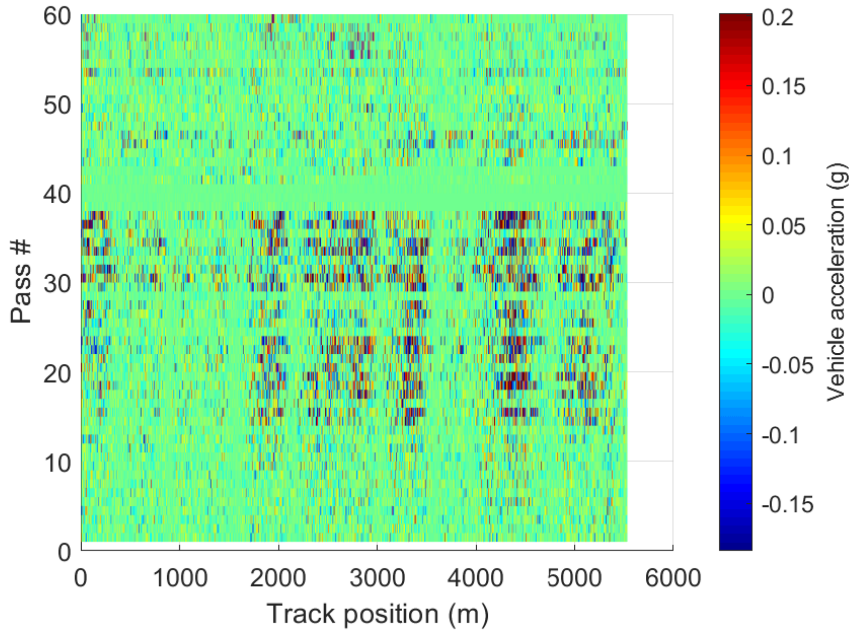

Figure A7: Uni-axle acceleration signals of LRV 4306 in region 8, 2015
